# Intermittent Frontal Rhythmic Discharges as an Electroencephalogram Biomarker of Acute SARS-CoV-2 Infection-Associated Encephalopathy in Children

**DOI:** 10.7759/cureus.19149

**Published:** 2021-10-30

**Authors:** Abdulhafeez Khair

**Affiliations:** 1 Neurology, Nemours Children's Health, Thomas Jefferson University, Wilmington, USA

**Keywords:** biomarker, frontal intermittent rhythmic discharges, eeg, encephalopathy, pediatric covid-19

## Abstract

Data on neurological sequelae of COVID-19 infection in children are sparse. Neurotropic and neuroinvasive potentials of the SARS-CoV-2 virus are a matter of ongoing scientific debate and not yet well understood. Most of the reported symptoms are nonspecific including headache, encephalopathy, weakness, and as a part of multisystem inflammatory response syndrome. Few observational studies have reported acute encephalopathy to be one of the neurological manifestations of COVID-19 infection, mostly in adults. A little is known about epileptogenesis or electroencephalogram (EEG) findings in this limited cohort of pediatric patients. We report a 17-year-old female with type 1 diabetes mellitus (DM), who presented with two weeks history of intermittent headaches, followed by a one-day history of acute change in behavior in the form of prolonged staring, decreased speech, confusion, and alternating periods of agitation and sleepiness. No fever or respiratory symptoms. Her blood glucose was normal. Brain MRI was unremarkable. Cerebrospinal fluid (CSF) studies showed 1000 RBCs, no WBCs, normal glucose/protein, negative culture, and negative infectious PCR, and autoimmune panels. She was found to be positive for SARS-CoV-2 PCR with negative IgG. Her EEG showed remarkable background slowing and frequent frontal intermittent rhythmic discharges. She was managed with high-dose steroids with the full clinical recovery of all symptoms at discharge, as well as normalization of subsequent EEG studies. We hypothesize there may be some specific seizure characteristics or EEG patterns in patients with pediatric COVID-19 infection and concomitant acute encephalopathy. It is perhaps reasonable to obtain EEG studies in children who test positive for SARS-CoV-2 and report central neurological symptoms. Long-term follow-up of this cohort of patients will be helpful to understand the clinical significance and implications of such neurophysiological studies.

## Introduction

Neurological symptoms associated are infrequently reported in the subset of pediatric patients with acute COVID-19 infection [1]. Few studies highlighted the potential isolation of SARS-CoV-2 PCR from the cerebrospinal fluid (CSF) of some patients; however, the full understanding of potential neuro-invasive characteristics of the virus is lacking [2]. Other non-invasive, neurotropic pathological mechanisms appear to continue to a various range of neurological symptoms [3]. Neurological symptoms of COVID-19 infection have been reported in about nearly 35% of adult patients and less than 20% of pediatric patients [4]. The incidence of neurological symptoms is higher in the cohort of pediatric multi-system inflammatory syndrome temporally associated with COVID-19 infection (PIMS-TS) [5]. The majority of reported symptoms are rather nonspecific. Nevertheless, acute encephalopathy with or without ongoing encephalitis has been reported in a few children [6]. The presence of a specific electroencephalogram (EEG) signature of acute COVID-19 encephalopathy is yet to be determined.

## Case presentation

A 17-year-old female with a known history of type I diabetes mellitus (DM) and hypercholesterolemia presented with an acute alteration of mental status. She was in her usual state of good health until her mother reported that she started complaining of headaches for two weeks. These headaches were intermittent and associated with a feeling of neck muscle tightness. She tried taking calcium and magnesium supplements as well as using topical oils to ease the pain. Then on one day morning, the mother found her staring and not speaking with minimal responsiveness to verbal and tactile stimulation. No associated eye-rolling, facial twitching, tongue biting, body posturing, or extremity shaking movements were noted. She appeared to be breathing comfortably but she continued to be confused for more than an hour, which ultimately prompted the family to bring her to the emergency department. Otherwise, there were no reported recent infections, fever, vomiting, weakness, or seizures. She was noted to be drinking more water than usual but her blood glucose checks at home were within the usual normal range. The night before the presentation she received an extra dose of Insulin as her home urine test showed some extra ketones. Overall, her diabetes control was described as suboptimal. A month before this presentation her HbA1C was 12.7. She had to be hospitalized five years prior due to acute diabetic ketoacidosis (DKA).

Upon evaluation in the emergency department, she was noted to be very confused and lethargic. Her vital signs were appropriate, and she was breathing spontaneously with no respiratory support. She was slow to respond to questions and unable to cooperate with simple task requests. Her remote memory was impaired. Her Glasgow coma scale was estimated to be 12. No cranial nerve palsies, motor weakness, or sensory impairment could be elicited. She was able to ambulate with her hands being held, but she had a slow, hesitant, wide-based gait. Her blood glucose was initially 252 mg/dL and mild acidosis with serum Ph of 7.24 needing an insulin bolus. Her measures serum osmolality was 283 mmol/kg. No other electrolyte imbalance was noted in her blood work. Extended drug screening was negative. Head CT without contrast was obtained and was unremarkable. She received intravenous fluids, but she remained confused and occasionally agitated. Testing for SARS-CoV-2 PCR was negative from nasal swab samples. The concern for possible COVID-19 attributed multisystem inflammatory syndrome (MIS-C) was entertained but work up including hematological and cardiac evaluations were not consistent with MIS-C.

**Figure 1:**
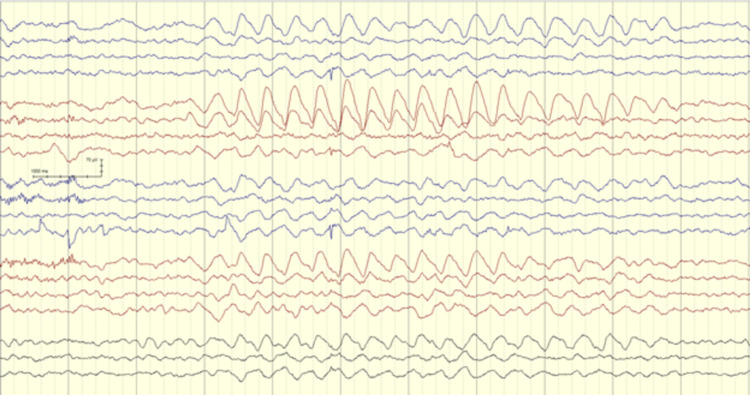
EEG showing asymmetrical FIRDA with right-side dominance FIRDA - Frontal Intermittent Rhythmic Delta Activity

**Figure 2:**
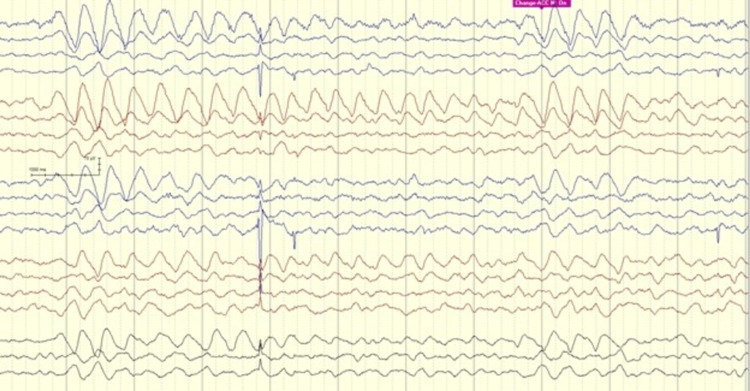
EEG showing recurring frontal intermittent rhythmic delta activity

With her DM history and given the remarkable change in sensorium, the neurology team was involved in the evaluation of encephalopathy and possibly cerebral edema. Non-contrast brain magnetic resonance imaging (MRI) including diffusion-weighted sequences, along with dedicated venography sequences (MRV) were all unremarkable. Lumbar puncture was performed with a normal CSF profile including negative screening for autoimmune encephalopathy antibody panel. EEG was performed to assess her egress of encephalopathy and eliminate the possibility of clinical or electrographic seizures. An overnight EEG showed a poorly formed posterior dominant rhythm of 8 Hz with superimposing low amplitude fast beta activity with anterior voltage predominance. Rhythmic runs of intermittent, yet consistent, dominantly right frontal slow theta and delta waves were noted throughout the record (Figures 1, 2).

There was no change in the EEG pattern in response to sensory or photic stimulation. Those discharges were consistent with Frontal Intermittent Rhythmic Delta Activity, also known as FIRDA. No other distinct epileptiform or ictal discharges were seen. It is worth noting that the Patient did not receive any sedative or pain medications at the time of those evaluations.

The patient was admitted to the hospital for two weeks, out of which eight days were spent in the pediatric intensive care unit for close monitoring of clinical progress and metabolic management. She developed a low-grade fever maxed at 100.8 F but all infectious workup was negative. She received a trial of high-dose methylprednisolone for five days for presumed immune-mediated encephalopathy with no tapering course. During her second week of hospitalization, she had a transient increase in her need for respiratory support needing high flow oxygen for two days. Chest x-ray at the time was interpreted as suggestive of nonspecific viral pneumonitis. Her mental status and behavior continued to improve throughout her hospitalization. Her neurological exam upon discharge was completely unremarkable. Post-discharge EEG which was done about three weeks after her first EEG was normal as well. As the above evaluations were thought to be non-diagnostic, the diagnosis of COVID-19 encephalopathy was concluded.

## Discussion

Recognition and understanding of neurological symptoms of pediatric COVID-19 infection have improved with advancing basic and clinical knowledge of SARS-CoV-2 infection characteristics. Children appear to represent less than 10% of the total case cohort, and about one-third of them have reported headaches as the most commonly observed neurological symptom [7]. A subset of children exhibit symptoms of acute encephalopathy such as disrupted sleep cycle, altered behavior, fluctuating level of consciousness, seizures, or upper motor neuron signs [8]. Full understanding of the pathophysiological process behind pediatric COVID-19 attributed encephalopathy is lacking, but hematogenous dissemination, direct brain invasion, secondary hypoxic brain injury, angiotensin-converting enzyme dysfunction, autoimmune-mediated neurotoxicity, and inflammatory cascade pathways activation are presumed contributing mechanisms [9]. Interestingly, the majority of patients have elevated CSF protein indicating some degree of blood-brain barrier disruption, but SARS-CoV-2 cannot usually be isolated from CSF [10]. The commonest brain neuroimaging findings are acute and subacute infarcts, leptomeningeal enhancement, microbleeds, and hyperintense signal abnormalities [11].

Although EEG is often used as a part of a neuro-diagnostic evaluation of patients with altered mental functions including those with COVID-19 encephalopathy, only a few studies have reported any particular pattern of EEG abnormalities. Reviews published earlier in the course of the current pandemic concluded that EEG findings are nonspecific and no pattern could be found [12,13]. Nevertheless, EEG findings do usually correlate with clinical severity of coma or mental status impairment and can thus offer valuable prognostic guidance. In half of the adult patients who remained comatose after weaning of all sedatives, the alpha coma pattern was a marker of a more grim prognosis or rather prolonged recovery [14]. Additionally, EEG background reactivity, in particular, can help to prognosticate neurological outcomes in patients with COVID-19-induced hypoxic-ischemic encephalopathy [15].

Background slowing is the most dominant EEG signature in various reports. In a cohort of 23 adult patients with COVID-19 encephalopathy, 17 had diffuse background slowing but none had any ictal discharges [10]. Pasiti et al. reported a group of 13 adult patients, all of them had mixed theta and delta 4-8 Hz slowing, among them four had consistent bifrontal slowing [16]. In 10 adult patients with severe encephalopathy reported by Canham, widespread delta slowing with mild anterior emphasis was recorded [17]. Flamand et al. reported an 80 years old patient whose EEG showed distinct 1-1.5 seconds periods of triphasic discharges consistent with toxic or metabolic encephalopathy [18]. Vellieux et al. reported two young adults with symmetric yet monomorphic, diphasic, delta slow waves with dominant frontal projection [19]. FIRDA is more commonly encountered in the neurocritical care setting and may indicate an associated vascular territorial injury [20]. The systemic review by Antony et al. of 617 patients concluded that frontal EEG patterns were noted in about one-third of patients and the authors suggested incorporating this pattern as a biomarker of COVID-19 encephalopathy [21].

The epileptogenic potential of the SARS-CoV-2 virus is a subject of active research. Conclusive evidence that COVID-19 infection directly generates cortical excitability is lacking. However, contributing etiologies to seizure emergence in the pediatric population are thought to include high fever, hypoxic insult due to severe respiratory compromise, and multi-organ dysfunction [22]. The overall prevalence of either generalized or more rarely focal EEG epileptiform discharges is estimated to be around 20% in total patients with COVID-19 encephalopathy [23]. Patients with pre-existing epilepsy and comorbid neurological conditions appear to be at a higher risk of having ictal or interictal epileptiform discharges [24]. To our knowledge, detection of isolated electrographic seizures without preceding or following clinical seizures is exceptionally rare [25,26]. However, a more recent review by Lin et al. reported that up to 4% of clinically ill patients may have evidence of non-convulsive status epilepticus in EEG without associated clinical seizures, which highlights both the importance and underutilization of EEG in critically sick COVID-19 patients [27]. Of note, despite the presence of an overall 1% incidence of acute symptomatic seizures as a part of COVID-19 infection symptomatology, the incidence of short and long-term epilepsy in those patients is not yet known [28].

Limitations and disruption of EEG resources availability to COVID-19 patients with neurological or critical symptoms have been noted across different health systems [29,30]. Unfortunately, and following a long history of disparity and inequality, children's access to EEG services has been more preferentially jeopardized [31]. This has contributed significantly to the rarity of published EEG data in COVID-19 infected children [32].

## Conclusions

Pediatric COVID-19 encephalopathy is a rare, yet serious clinical phenotype variant of SARS-CoV-2 infection responsible for the current worldwide pandemic. EEG can elicit both non-diagnostic and potentially specific characteristics. Whether focal, frontally dominant, periodic (as recorded in our reported patient), and continuous background slowing represents a specific EEG signature biomarker for acute COVID-19 is to be determined. It appears to be plausible to screen children with clinical encephalopathy and those EEG findings for COVID-19.
